# Diagnostic Advantages of Proximal Sacroiliac Joint Injections for Relief of Mechanical Back Pain: A Retrospective Observational Cohort Study

**DOI:** 10.7759/cureus.116007

**Published:** 2026-09-09

**Authors:** Alan I Shain, Zhiyan Yang, William Xiang, Sohyun Kang, Jaspal R Singh

**Affiliations:** 1 Physical Medicine and Rehabilitation, Northwell Health, New York, USA; 2 Physical Medicine and Rehabilitation, Weill Cornell Medicine and Columbia University/New York-Presbyterian Hospital, New York, USA

**Keywords:** contrast dye, distal approach, fluoroscopy, interventional pain management, intra-articular injection, low back pain, lower one-third joint technique, proximal approach, sacroiliac joint, upper one-third joint technique

## Abstract

Background

The sacroiliac joint (SIJ) is a common source of mechanical low back pain (LBP). Standard fluoroscopy-guided intra-articular injections typically target the distal (lower one-third) SIJ, but some patients fail to achieve relief, raising the possibility that the distal approach may not adequately access clinically relevant pain generators in the proximal fibrous SIJ.

Objectives

The primary objective was to evaluate the immediate diagnostic efficacy of the proximal (upper one-third) SIJ injection approach -- defined as ≥50% visual analog scale (VAS) reduction within 30 minutes of injection (reflecting the local anesthetic's diagnostic response rather than therapeutic benefit) -- in patients with mechanical LBP unresponsive to prior distal SIJ injections. Secondary objectives included evaluating complete pain resolution, demographic associations (age and sex), and procedural safety.

Study design

This was a retrospective observational cohort study.

Setting

The study was conducted at Och Spine-Center for Comprehensive Spine Care, Department of Physical Medicine and Rehabilitation, Weill Cornell Medicine, New York-Presbyterian Hospital.

Methods

Twenty-two adult patients with mechanical LBP who previously failed to achieve therapeutic relief from technically adequate distal SIJ injections performed by outside providers underwent fluoroscopically guided proximal SIJ injections by a single interventional physiatrist. Procedures targeted the upper third of the SIJ using a therapeutic injection of corticosteroid and local anesthetic. Baseline and post-injection pain scores were recorded using the VAS within 30 minutes pre- and post-injection to measure the immediate diagnostic response of the anesthetic. The primary outcome was the proportion of patients achieving ≥50% pain reduction in this immediate response. Secondary outcomes included complete pain resolution, associations with age or sex, and complications.

Results

All patients underwent technically successful proximal SIJ injections as confirmed by intra-procedural contrast dye patterns. The baseline VAS score (mean ± SD) was 4.86 ± 2.75, and the post-injection score was 1.14 ± 1.42, yielding a mean pain improvement of 79.6% ± 25.8% (p < 0.001). Twenty patients (90.9%) achieved ≥50% reduction in pain (95% confidence interval: 70.8%-98.9%), and 10 patients (45.5%) experienced complete resolution. No complications were reported. There were no statistically significant differences in outcomes by age or sex.

Limitations

This study was retrospective, with short-term follow-up limited to immediate post-injection pain scores. Functional outcomes and longer-term pain relief were not assessed. Patients were referred from multiple providers, potentially introducing selection bias. All proximal injections were performed by a single provider, potentially introducing performance bias. Future prospective studies should evaluate long-term outcomes, patient-reported functional measures, and relevant spino-pelvic parameters.

Conclusions

Proximal SIJ injections yielded high rates of immediate pain relief in patients who initially failed technically adequate distal injections. These findings suggest anatomical and diagnostic advantages of the proximal approach that may identify SIJ pain generators not accessed by distal injections. Prospective controlled studies are needed to evaluate whether the proximal approach warrants earlier consideration in the mechanical SIJ pain treatment algorithm.

## Introduction

The sacroiliac joint (SIJ) is a common cause of mechanical low back pain (LBP), estimated to be implicated in 10% to 27% of cases [1-3]. The pathophysiology of SIJ pain can stem from ligamentous laxity and strain (pregnancy, connective tissue disorders), abnormal load distributions on the SIJ (muscle weakness, altered gait mechanics), alterations in global spino-pelvic alignment (such as variations in pelvic incidence, sacral slope, and pelvic tilt), degenerative changes (osteoarthritis), trauma, repetitive stress, and overuse (excessive or misguided athletic activities), among other factors [4-7]. When conservative management fails, an SIJ injection, consisting of a short-acting anesthetic and a corticosteroid, is a common intervention for both diagnostic and therapeutic purposes. Practitioners most frequently opt for the distal (lower one-third) approach for SIJ injections because of its relative ease of access, more conventional technique, and well-defined anatomical landmarks [8]. This approach traditionally targets the caudal portion of the joint, the synovial compartment, as this area is considered the primary site for effective access under fluoroscopic guidance [1,6,8,9].

Technical success in SIJ injections refers to accurate intra-articular needle placement with appropriate contrast spread, while therapeutic success reflects clinically meaningful pain relief, irrespective of fluoroscopic findings. Distal injections are typically judged to be technically successful when contrast outlines the joint space and migrates cephalad along the SIJ line [2,6,8]. However, technical success does not necessarily predict therapeutic benefit. Recent literature has emphasized ≥50% pain relief or reaching a minimum clinically important difference on the visual analog scale (VAS) or numeric rating scale (NRS) as more relevant outcome measures [2,6,8,10-12].

Despite technically successful distal injections, some patients do not experience sustained pain relief, particularly at longer follow-up [4,13,14]. While contrast delivery to the superior SIJ has been associated with greater pain reduction at two weeks, eight-week outcomes appear unrelated to contrast patterns, suggesting deeper anatomical drivers of chronic SIJ pain [13]. These drivers may be related to anatomical barriers, such as joint space narrowing, osteophyte formation, or limited access to proximal pain generators in the fibrous portion of the SIJ [2,4,6,8,9,13,15,16]. Electrophysiologic and histologic studies have shown a high concentration of nociceptive fibers in the proximal fibrous SIJ capsule, particularly within the interosseous and posterior sacroiliac ligaments, which are less easily accessed via the distal approach [4,17,18]. These interosseous and posterior sacroiliac ligaments help anchor the proximal SIJ, and the interosseous ligaments bear the highest strains during spinal motion [5]. Together, these structures concentrate the greatest density of nociceptive fibers within the joint, making anesthetic delivery to this region particularly likely to yield a meaningful diagnostic response [4,17,18]. These anatomical considerations provide a rationale for targeting the proximal fibrous region when assessing SIJ‑mediated pain.

As a result, the proximal (upper one-third) approach has gained attention for its ability to more directly target these ligamentous structures [4,6,8,17,19,20]. Though initially considered a secondary or salvage method, comparative trials now show similar technical success rates between the distal and proximal approaches (95.6% vs. 93.3%, respectively), with no significant difference in contrast spread [8]. Importantly, the proximal approach demonstrated a 90% technical success rate even when used after distal failure [6]. The only domains demonstrating inferior performance were procedural time and comfort, both likely modifiable with increased provider experience [8]. Historically, studies have focused on contrast spread or long-term corticosteroid response; however, the immediate anesthetic diagnostic response provided by the proximal approach has received less attention and may also reflect successful targeting of clinically relevant SIJ pain generators.

The purpose of this study is to evaluate the immediate diagnostic efficacy within 30 minutes post-procedure of the proximal approach in alleviating mechanical LBP in patients who failed to experience adequate pain relief following initial distal SIJ injections. This study aims to provide evidence supporting the proximal approach as an effective diagnostic tool to improve identification of SIJ-mediated pain, particularly for patients in whom the distal technique proves insufficient.

## Materials and methods

Study design

This retrospective cohort study was conducted at the Och Spine Center for Comprehensive Spine Care at NewYork-Presbyterian/Weill Cornell Medicine. The study aimed to assess the clinical outcomes of patients with mechanical LBP who failed to achieve adequate pain relief from SIJ injections via the distal approach and were subsequently treated using the proximal approach under fluoroscopic guidance. The study was approved by an institutional review board (IRB protocol #24-12028284), with waivers of informed consent and of HIPAA authorization granted due to the retrospective nature of the review and deidentification of all patient information collected. The authors declare no conflicts of interest. This research received no external funding.

Patient selection

Patients were referred from multiple clinical sites after failing to achieve pain relief with initial distal SIJ injections, which had been performed by various providers across different pain management centers. In contrast, all proximal SIJ injections were performed by a single experienced interventional physiatrist (J.R.S.) at Och Spine Center for Comprehensive Spine Care at New York-Presbyterian/Weill Cornell Medicine. 

Eligible patients were identified via manual review of electronic medical records of patients treated between January 1, 2023, and March 14, 2025. Patients were required to be between 18 and 75 years of age, demonstrate ≥3 positive provocative SIJ maneuvers (FABER test, Gaenslen’s test, compression test, thigh thrust test, Fortin finger test, and distraction test), and have underwent radiographic evaluation supporting a mechanical source of LBP without features suggestive of alternative causes such as malignancy, infection, or spondyloarthritis [21-23]. All provocative maneuvers were assessed by a single experienced interventional physiatrist (J.R.S.) upon patient arrival for the proximal SIJ injection procedure, prior to review of imaging findings or injection outcomes. All eligible patients failed to achieve pain relief following an initial fluoroscopically guided distal SIJ injection. This necessitated follow-up with an interventional physiatrist for persistent pain. These patients subsequently received a fluoroscopically guided proximal SIJ injection in our clinic. All patients had complete baseline and post-procedural pain data (VAS scores) recorded within 30 minutes before and within 30 minutes after the proximal injection.

Exclusion criteria included incomplete records, significant confounding conditions (e.g., infections, malignancy, poorly controlled diabetes, coagulopathy, pregnancy), known allergies to the injectate, and procedures performed outside the specified date range.

Procedural technique

All procedures were performed by the same interventional physiatrist under sterile conditions with fluoroscopic guidance. No sedation was used. Local anesthesia with 2% lidocaine was administered to the skin and subcutaneous tissue prior to needle insertion. Details pertaining to the distal approach, performed prior to referral by various outside physiatrists, were determined through chart review and were similar to the technique described by Hong et al. [8].

**Figure 1:**
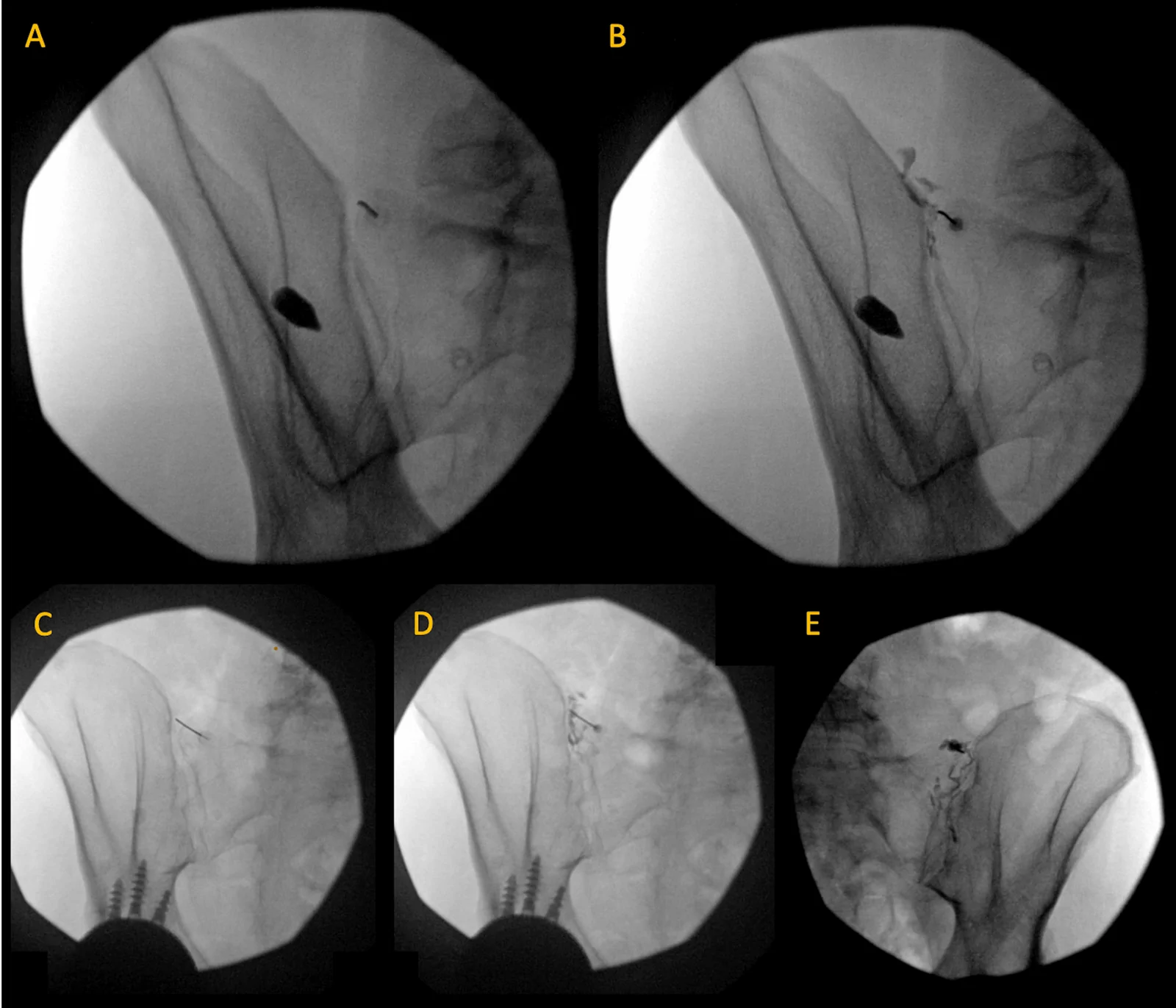
Fluoroscopic images demonstrating the proximal SIJ injection technique in two representative patients. (A, B) Images from the same patient demonstrating (A) initial fluoroscopic positioning under cephalad and contralateral oblique angulation exposing the proximal sacroiliac joint behind the iliac crest, followed by (B) needle placement within the upper third of the SIJ with contrast confirming cephalocaudal intra-articular spread. (C, E) Images from a different patient showing (C) needle entry into the proximal SIJ, (D) contrast outlining the joint following redirection around the iliac crest, and (E) dense cephalocaudal intra-articular contrast fill confirming complete cephalocaudal distribution within the fibrous joint space.

The proximal approach was performed as follows: the fluoroscope was tilted in the cephalad direction to square off the L5-S1 endplate. Then, the beam was contralaterally obliqued to expose the proximal portion of the SIJ, which can often be hidden behind the iliac crest. Ideal obliquity (typically 10° to 15°) was achieved when the space between the proximal lateral sacral ala and the iliac crest was optimized. The skin and subcutaneous tissues were anesthetized with 2% lidocaine. A 22-gauge Quincke spinal needle (Becton Dickinson, Franklin Lakes, NJ, USA) was inserted coaxially with the beam toward the upper third of the joint composing the fibrous portion of the SIJ. Once resistance from the iliac crest was encountered, the needle was maneuvered to enter the joint space. Contrast injection confirmed intra-articular spread in a cephalocaudal fashion without vascular uptake. Following confirmation, a mixture of 1.5-2.5 mL of 2% lidocaine and 40 mg triamcinolone was injected. Total fluoroscopy time was approximately 15 seconds per case. This technique is demonstrated in Figure 1, which shows representative fluoroscopic images from two patients undergoing proximal SIJ injection via this approach.

Data collection

Clinical and procedural data were manually extracted from the electronic medical records, including demographic information such as age and sex, baseline and post-injection VAS pain scores (recorded within 30 minutes before and after the procedure), and any documented complications. All data were de-identified and stored securely in REDCap (Vanderbilt University, Nashville, TN, USA), a HIPAA-compliant database. Each patient record was assigned a unique, randomized study ID without linkage to identifiable information.

Outcomes

The primary outcome was the proportion of patients achieving ≥50% reduction in pain based on the VAS score 30 minutes post-injection. Pain scores were extracted from standardized clinical documentation before and after the proximal SIJ injection. To further evaluate efficacy, pain scores were also categorized by therapeutic response: Complete (100%), Significant (70-99%), Moderate-to-High (50-70%), Moderate-to-Low (30-50%), Low (1-30%), and None (0%). Secondary outcomes included the presence of any documented complications (e.g., numbness, infection) and the association between demographic/clinical variables (e.g., age, sex) and degree of pain relief. Age-related differences were analyzed by dividing patients into younger and older groups based on the median age, determined after data collection.

Statistical analysis

The 95% confidence interval (CI) for the primary outcome was calculated using the Clopper-Pearson exact method (most appropriate for binomial proportions in small samples). Baseline and post-injection VAS scores were compared using a paired t-test. The normality of the paired differences between scores was assessed using the Shapiro-Wilk test. VAS improvement in pain was compared between male and female patients, as well as between younger (<38 years) and older (≥38 years) patients, using the Mann-Whitney U test. Values were reported as mean ± standard deviation (SD) where appropriate or as median (first quartile Q1 - third quartile Q3). As by convention, the smaller U statistic value was reported for the Mann-Whitney U test. A p-value <0.05 was considered statistically significant. Analyses were performed using R statistical software version 4.6.1 (R Foundation for Statistical Computing, Vienna, Austria). Researchers may contact the corresponding author for further information regarding the analytic approach.

## Results

A total of 22 patients (12 female patients (55%), 10 male patients (45%)) who failed to achieve significant pain relief following distal SIJ injections were included. The median age of 38 years was used to divide patients into older or younger groups. Technical success, defined as satisfactory intra-articular contrast spread during the proximal SIJ injection, was achieved in all 22 cases (100%). 

The baseline VAS score (mean ± SD) was 4.86 ± 2.75. Following the proximal injection, the post-injection VAS score decreased to 1.14 ± 1.42, corresponding to a mean VAS improvement of 79.6% ± 25.8% (Table 1). Ten patients (45.5%) reported complete resolution of pain, and an additional 10 (45.5%) patients achieved significant or moderate-to-high resolution of pain without complete resolution (Table 2). A total of 20 patients (90.9%) achieved the primary outcome of clinically meaningful improvement (95% CI: 70.8%-98.9%), defined as ≥50% reduction in VAS score (Table 2). Only one patient (4.5%) reported no improvement at all (Table 2).

**Table 1:** Patient demographics and baseline characteristics. VAS improvement reflects the relative reduction in VAS score from baseline. Younger patients were defined as those younger than the cohort median age (<38 years), and older patients were defined as those aged 38 years or older (≥38 years). Q1 and Q3 represent the first (25th percentile) and third (75th percentile) quartiles, respectively. ^a^Values = mean ± SD. VAS = visual analog scale; n = number of patients.

Characteristic	Value
Total patients (n)	22
Median age, years (Q1-Q3)	38 (29-56)
Male/Female (n)	10/12
Younger patients (n)	11
Older patients (n)	11
Baseline VAS^a^	4.86 ± 2.75
Post-injection VAS^a^	1.14 ± 1.42
VAS improvement (%)^a^	79.6 ± 25.8

**Table 2:** Categorized patient outcomes based on VAS improvement after proximal SIJ injection in 22 patients. The primary outcome (≥50% improvement) encompassed the Complete, Significant, and Moderate-to-High groups. VAS = visual analog scale; n = number of patients.

VAS Improvement	Number of Patients (N)	Proportion of Cohort (%)
Complete (100%)	10	45.5
Significant (70-99%)	7	31.8
Moderate-to-high (50-70%)	3	13.6
Moderate-to-low (30-50%)	1	4.5
Low (1-30%)	0	0
None (0%)	1	4.5
Primary outcome (≥50% improvement)	20	90.9

The results of the statistical analyses are summarized in Table 3. Statistical analysis confirmed a significant reduction in pain following the proximal injection (paired t-test, p < 0.001), and the paired differences met normality assumptions (Shapiro-Wilk, p = 0.204). In the subsequent subgroup analyses comparing outcomes by age and gender, at least one subgroup violated the assumption of normality; therefore, the Mann-Whitney U test was used. No statistically significant differences in VAS improvement were observed between sex groups (two-tailed Mann-Whitney U = 53.0, p = 0.652) or between younger (<38 years) and older (≥38 years) patients (two-tailed Mann-Whitney U = 49.5, p = 0.468).

**Table 3:** Statistical analysis of clinical outcomes. ­Row 1 compares paired pre- and post-procedure VAS scores within the same patients, whereas rows 2 and 3 compare the percentage improvement in VAS scores between gender and age groups, respectively. Values are presented as mean ± SD for the paired t-test comparison and median (Q1-Q3) for Mann-Whitney U test comparisons. Q1 and Q3 represent the first (25th percentile) and third (75th percentile) quartiles, respectively. The median age (38 years) was used to define younger and older patient groups. A p-value <0.05 was considered statistically significant. SD = standard deviation; U = Mann-Whitney U statistic (by convention, reporting the smaller U statistic value); t = paired t-test statistic; VAS = visual analog scale; n = number of patients.

Comparison	Group 1	Group 2	Test Used	Parameter Values	p-Value
Baseline vs. post-injection VAS (mean ± SD)	Baseline (n = 22): 4.86 ± 2.75	Post-injection (n = 22): 1.14 ± 1.42	Paired t-test	t = 9.05	<0.001
VAS improvement male patients vs. female patients, median (Q1-Q3)	Male patients (n = 10): median 80.0% (77.8%-100%)	Female patients (n = 12): median 88.9% (55%-100%)	Mann-Whitney U test	U = 53.0	0.652
VAS improvement younger vs. older, median (Q1-Q3)	Younger <38 (n = 11): median 100.0% (75%-100%)	Older ≥38 (n = 11): median 77.8% (60%-100%)	Mann-Whitney U test	U = 49.5	0.468

Importantly, no procedural complications occurred. There were no reported cases of infection, neurologic injury, or adverse reactions to the injectate, supporting the safety of the proximal SIJ injection technique when performed under fluoroscopic guidance.

## Discussion

This retrospective study demonstrated that the proximal SIJ injection approach can provide substantial pain relief within 30 minutes post-injection, even in patients who failed to receive immediate relief from distal injections, consistent with the proximal approach’s role as an effective diagnostic, rather than purely therapeutic, intervention. Among our cohort of 22 patients with mechanical LBP who failed distal SIJ injections, 45.5% experienced complete pain resolution, while an additional 45.5% achieved a clinically meaningful improvement without full resolution. In total, 90.9% met the primary outcome of ≥50% improvement in VAS score, and only one patient failed to show any improvement. These results highlight the proximal technique’s meaningful diagnostic benefit in patients unresponsive to the distal approach, as demonstrated by the significant reduction in mean VAS scores following injection. However, these findings must be interpreted as measuring the immediate local anesthetic diagnostic response, not long-term therapeutic benefit or corticosteroid efficacy, as the 30-minute assessment specifically captures anesthetic effect [19,21]. While these findings of substantial pain relief are compelling, alternative explanations including regression to the mean, placebo response, and non-blinded subjective VAS assessment should be acknowledged and are discussed further in the limitations. Despite these considerations, the consistent response observed following proximal injection provides evidence that the proximal approach may have an anatomical advantage in identifying the proximal SIJ as a pain generator.

Our cohort consisted of patients who had no meaningful clinical improvement despite technically successful distal injections and were referred for persistent pain. Nearly all then demonstrated a positive response to the proximal technique, supporting SIJ pain as the underlying diagnosis. This pattern strongly suggests that the proximal approach engages relevant anatomy missed by the distal approach. Delivered through an oblique trajectory, the proximal approach provides access to the upper third of the SIJ, where the interosseous and posterior sacroiliac ligaments are located. These fibrous structures are rich in nociceptive fibers and frequently implicated in SIJ-mediated pain [4,6,8,17,19,20]. In contrast, the distal approach targets the caudal synovial compartment and may fail to reach these more proximal structures, particularly in patients with joint space narrowing or osteophytic obstruction [2,4,6,8,13,15]. Prior research by Scholten et al. has shown that contrast spread does not always correlate with sustained pain relief, suggesting that technical success alone does not guarantee therapeutic success [13]. This likely explains why patients in our study, despite technically adequate distal injections, experienced substantial relief only following the subsequent proximal approach. These anatomical and clinical considerations may account for the high rate of immediate anesthetic response observed in our cohort, despite prior SIJ injections. Collectively, the findings support the proximal approach as a more promising diagnostic intervention in patients with prior distal non‑response.

Our findings are consistent with prior studies. Park et al. demonstrated 90% technical success with the proximal approach in patients with technically unsuccessful distal injections, and Hong et al. found the proximal approach non-inferior to the distal technique in achieving intra-articular contrast spread, with similar technical success (95.6% vs. 93.3%) and no increase in complications [6,8]. However, our study differs in that distal failure was defined not by technical difficulty, but by lack of therapeutic response, reflecting a patient-centered rather than procedure-centered definition of success.

There was a clinically significant difference in pain outcomes (mean ± SD) between baseline and post-injection scores in our study (VAS 4.86 ± 2.75 to 1.14 ± 1.42), which parallels the differences reported by Park et al. (NRS 7.1 ± 1.1 to 1.1 ± 1.2) and Hong et al. (NRS proximal group: 6.97 ± 1.41 to 2.24 ± 1.87; distal group: 6.59 ± 1.37 to 2.52 ± 1.97) [6,8]. Notably, the baseline pain scores in our cohort were more moderate (VAS 4.86 ± 2.75) than those reported in Park et al. and Hong et al., which may reflect some partial benefit from the distal injection, though not sufficient to meet clinically meaningful thresholds. However, neither study reported the proportion of patients achieving a ≥50% reduction or meeting a minimum clinically important difference. We chose to report the percentage of patients with ≥50% pain improvement in addition to average VAS scores, aligning with other SIJ injection literature [24]. This approach accounts for the subjective nature of NRS/VAS and better reflects individual-level therapeutic success, particularly in smaller samples. Subgroup analyses in our study found no significant differences in improvement by age or sex, although these findings should be interpreted cautiously given the small subgroup sizes and exploratory nature of the analyses.

Importantly, both Park et al. and Hong et al. present the proximal approach as a salvage or non-inferior alternative to the distal approach. In contrast, our findings suggest that the proximal approach may merit consideration earlier in the treatment algorithm, given its precise anatomical targeting and promising immediate diagnostic yield. Emphasis on patient‑reported pain relief and immediate diagnostic response, rather than on technical success alone, aligns with a broader trend in interventional care that prioritizes meaningful symptom relief over solely relying on imaging-based benchmarks [2-4,25]. Current clinical guidelines do not provide a standardized algorithm for when to utilize the proximal approach, either initially or following failed distal injections [2-4,9,21,25]. The 2025 McCormick et al. practice guidelines, a recently published multispecialty, international consensus on SIJ complex pain, recognize the proximal approach as one alternative to consider when inferior joint access is not feasible, assigning it a Grade C recommendation based on low-to-moderate certainty of evidence [9]. These guidelines within the same recommendation additionally acknowledge a middle-portion approach to the SIJ, targeting a distinct anatomical landmark as another consideration when the conventional distal approach is not feasible [9,16]; however, the existing literature that describes this technique or similar techniques targeting the middle portion of the joint has focused on technical access success rather than clinically meaningful pain relief [16,26]. This recommendation does not extend to a standardized algorithm for the proactive use of the proximal approach as an alternative injection approach. The gap between a Grade C recommendation and a standardized algorithm limits the practitioner’s ability to personalize treatment based on the most probable anatomical sources of pain. Our findings add to the body of evidence that the proximal approach may warrant prospective evaluation as an earlier diagnostic option in appropriately selected patients, and as an especially valuable option in those with distal non‑response.

Further supporting this perspective, prior studies have shown that periarticular SIJ injections more effectively target the richly innervated posterior ligamentous structures compared to traditional intra-articular distal techniques [19,27-29]. Hansen et al. wrote a systematic review evaluating the therapeutic efficacy of SIJ interventions, which concluded that the evidence supporting periarticular injections with local anesthetic, steroids, or botulinum toxin was limited or poor, and similarly rated the evidence for intra-articular steroid injections as limited [30]. Despite the limited evidence for both procedures, clinical guidelines have continued to support intra-articular injections due to their strong diagnostic specificity [25]. The intra-articular proximal approach bridges this divide, offering both robust diagnostic value and more accurate therapeutic targeting of the fibrous SIJ anatomy responsible for chronic pain.

Hong et al. reported a significantly longer procedure time for the proximal approach in their cohort, which they attributed to greater operator familiarity with the conventional distal technique and the greater distance between the skin and the proximal target compared with the distal joint space [8]. In contrast, in our series, proximal injections were typically completed with short fluoroscopy times (approximately 15 seconds) by an interventional physiatrist experienced with the proximal approach and were often technically straightforward, suggesting that with increased familiarity, this technique may offer favorable procedural efficiency alongside its promising immediate diagnostic yield for SIJ‑mediated pain. However, our study did not include a contemporaneous distal control group, so formal comparisons of procedural speed or diagnostic accuracy between approaches cannot be made.

While the short-term results were highly encouraging, the durability of symptom relief following proximal SIJ injection remains unknown. Longer-term outcome studies are needed to confirm sustained benefit and further validate the proximal technique as a first-line intervention.

This study has several limitations. Regarding study design and methodology, a retrospective analysis cannot establish causality and is subject to documentation bias. The total number of charts screened and patients excluded at each stage were not prospectively recorded, potentially introducing selection bias. Patients were referred from multiple outside providers, further introducing selection bias and precluding independent verification of the technical adequacy of the index distal SIJ injections via chart review alone. All proximal injections were performed by a single experienced provider, improving consistency, but potentially limiting generalizability and introducing performance bias. However, distal injections were performed by multiple providers, introducing technical variability that may have contributed to some treatment failures independent of any true anatomical limitation. Additionally, procedure time was not directly compared between approaches under controlled conditions with equally experienced providers. Prospective studies with standardized injection protocols and centralized procedural oversight would be necessary to address these limitations. 

Additional limitations relate to outcome assessment and statistical considerations. Pain outcomes were assessed only in the short term (within 30 minutes before and after procedures), without follow-up to evaluate sustained benefit. Functional measures such as the Oswestry Disability Index were also not collected, limiting assessment of the broader clinical impact [11,31]. Although the sample size was adequate to demonstrate significant improvement, it was not large enough to support robust subgroup analyses. Age subgroup analyses utilized a post-hoc median split rather than a pre-specified threshold, which may introduce instability in our findings and limit comparability with other studies. Furthermore, the observed magnitude of improvement should be interpreted in light of several potential confounds. These include regression to the mean, given that patients likely presented during a symptomatic flare (after failed distal SIJ injections), placebo response to a second procedural intervention (with false-positive rates of 19-22% reported for single SIJ diagnostic blocks [21]), and the non-blinded subjective nature of VAS assessment in the absence of a sham or comparator arm. Future prospective studies should aim to incorporate larger sample sizes, longer-term follow-up, functional outcome measures, relevant spino-pelvic parameters, and standardized procedural protocols to strengthen the evidence base.

## Conclusions

The proximal SIJ injection technique yielded high rates of immediate pain relief in patients with persistent pain despite technically successful distal injections. This proximal approach appears to offer anatomical and diagnostic advantages over the distal approach, representing a promising option for image-guided intervention in mechanical SIJ pain. Prospective controlled studies are needed to compare proximal and distal approaches, assess longer-term outcomes, and determine whether the proximal approach merits consideration earlier in the treatment algorithm beyond its current role as a salvage maneuver.
